# Medicine Targeting Epithelial-Mesenchymal Transition to Treat Airway Remodeling and Pulmonary Fibrosis Progression

**DOI:** 10.1155/2023/3291957

**Published:** 2023-11-29

**Authors:** Hongjuan He, Xiaoyan Ji, Lihua Cao, Zhenzhen Wang, Xiaoyu Wang, Xiu-Min Li, Mingsan Miao

**Affiliations:** ^1^Academy of Chinese Medical Sciences, Henan University of Chinese Medicine, Henan, Zhengzhou 450046, China; ^2^Department of Otolaryngology, Microbiology and Immunology, New York Medical College, New York, NY 10595, USA

## Abstract

*Objective*. Dysregulation of epithelial-mesenchymal transition (EMT) in the airway epithelium is associated with airway remodeling and the progression of pulmonary fibrosis. Many treatments have been shown to inhibit airway remodeling and pulmonary fibrosis progression in asthma and chronic obstructive pulmonary disease (COPD) by regulating EMT and have few side effects. This review aimed to describe the development of airway remodeling through the EMT pathway, as well as the potential therapeutic targets in these pathways. Furthermore, this study aimed to review the current research on drugs to treat airway remodeling and their effects on the EMT pathway. *Findings*. The dysregulation of EMT was associated with airway remodeling in various respiratory diseases. The cytokines released during inflammation may induce EMT and subsequent airway remodeling. Various drugs, including herbal formulations, specific herbal compounds, cytokines, amino acid or protein inhibitors, microRNAs, and vitamins, may suppress airway remodeling by inhibiting EMT-related pathways.

## 1. Introduction

Airway remodeling refers to the progressive pathology of obstructive lung diseases, including asthma and chronic obstructive pulmonary disease (COPD) [1]. It is defined as the reorganization of the components of the airway walls due to chronic injury, leading to changes in structure and function. These repeated inflammatory stimuli influence the thickness of the airway walls of patients and gradually affect the whole bronchial tree. Airway remodeling is characterized by epithelial shedding, goblet cell hyperplasia between epithelial cells, airway wall thickening, epithelial fibrosis, vascular hyperplasia, and lumen stenosis. These characteristics can lead to an irreversible decline in lung function, severe restriction of airflow, and airway obstruction [2].

The main pathological features of asthma include airway inflammation and airway remodeling. Currently, most patients with asthma can manage their condition by well-established treatments such as inhaled corticosteroids (ICSs) and *β*2-adrenergic agonists. These therapies are considered first-line treatments because of their potent anti-inflammatory effects but are not effective for airway remodeling [3]. However, approximately 10% of patients with asthma are poorly managed and have an increased risk of hospitalization, which is associated with airway remodeling and airway constriction [4, 5]. Airway remodeling causes airflow limitations and airway obstruction that endanger the lives of patients with severe asthma. The rate of progression of these structural changes has been clinically intractable. Therefore, understanding the mechanism of airway remodeling will help to effectively control the changes in the early stages of asthma.

In recent years, it has been demonstrated that an essential mechanism of airway remodeling is the dysregulation of epithelial-mesenchymal transition (EMT) [2]. EMT is a complicated process related to tissue remodeling, in which epithelial cells lose their epithelial function of cell-cell adhesion and gradually transform into mesenchymal-like cells with the abilities of migration and invasion [6, 7]. During EMT, biomarkers of epithelial cells, such as E-cadherin, are repressed. In contrast, mesenchymal markers, including vimentin, matrix metallopeptidase (MMP) 7, MMP9, and *α*-SMA (alpha-smooth muscle actin), are upregulated. EMT is a novel clinical therapeutic target since it is activated in wound healing, cancer progression, and severe chronic airway diseases such as asthma and COPD [6].

EMT occurs through the stimulation of certain growth factors and is influenced by a variety of signalling pathways. For example, transforming growth factor-beta 1 (TGF-*β*1) may induce EMT in asthma, and it was shown to be increased in the bronchoalveolar lavage fluid of patients with asthma and was associated with increased airway thickness [8]. During airway remodeling, various immune cells and immune factors contribute to EMT.

At present, there are an increasing number of studies on herbal compounds or other medicines targeting EMT to inhibit the progression of airway remodeling and pulmonary fibrosis in patients with asthma and COPD. In this review, we describe the development of airway remodeling based on the connections between EMT signalling and immune factors. We also review the results of research on the use of herbal compounds, some amino acids, microRNAs, and vitamins to inhibit EMT-induced airway remodeling.

## 2. EMT-Induced Airway Remodeling

### 2.1. EMT and Airway Inflammation

**Figure 1:**
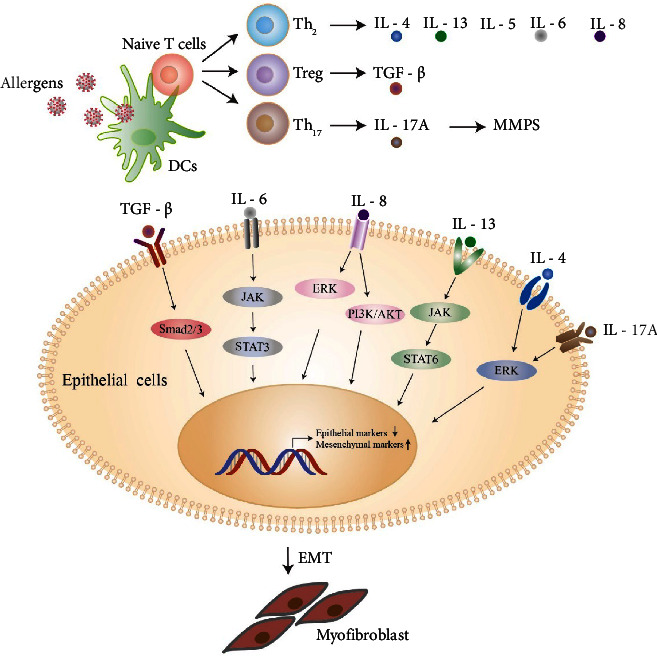
Overview of airway inflammatory cytokines and transcription factors that lead to EMT. T helper-type cells are stimulated by allergens, and Th2 or Th17 cytokines such as interleukin (IL)-4, IL-5, and IL-13 are secreted. Cytokines act as mediators that induce EMT. The growth factors secreted by immune cells can also interact with their receptors, activate transcription factors, and cause EMT.

Multiple cytokines and transcription factors are involved in the progression of EMT (Figure 1). Transforming growth factor-beta (TGF-*β*) is a critical growth factor that has been shown to contribute to EMT in several cell lines. Other growth factors, such as platelet-derived growth factor (PDGF), connective tissue growth factor (CTGF), fibroblast growth factor (FGF), and vascular endothelial growth factor (VEGF), were also shown to trigger EMT in lung diseases [9]. There is increasing evidence suggesting that tumour cells promote EMT by secreting inflammatory factors, including interleukin (IL)-6 and tumour necrosis factor alpha (TNF-*α*) [10]. Treatment of ovarian cancer with IL-8 has been shown to induce EMT, and there is evidence that it increases the invasiveness of cells, increases the expression of vimentin and snail, and downregulates the expression of E-cadherin [11]. Various cytokines, such as TNF-*α*, which are secreted by immune cells, also induce EMT [12]. The secretion of TGF-*β*1 and TNF-*α* was also reported to induce EMT in bronchial epithelial cells [13]. It has been shown that the induction of EMT by TGF-*β*1 in A549 cells may be enhanced by other cytokines, including IL-1, TNF-*α*, and IFN-*γ*, and there is evidence that the cells acquired fibroblast-like shapes and lost expression of E-cadherin [14]. These results suggest that inflammatory cytokines and transcription factors may play an important role in the progression of EMT.

These cytokines and transcription factors may induce EMT through various mechanisms via certain signalling pathways. Activation of TGF-*β* and TNF-*α* is regulated by TGF-*β* activated kinase-1 (TAK-1), which phosphorylates target proteins and may play an important role in the inflammatory accentuation of EMT [15]. IL-13 induces the production of collagen and airway fibroblasts in airway remodeling by stimulating the JAK/STAT6 and Erk/MAPK pathways [16]. IL-6 is associated with the STAT3 signalling pathway, which may be the mechanism for the induction of EMT in cancer [17]. According to studies on 16-HBE cells, IL-4 and IL-17 provide a chronic inflammatory environment that induces bronchial EMT. IL-4 and IL-17A synergize with TGF-*β*1 to enhance the capacity of TGF-*β*1 to induce EMT through the regulation of Erk1/2 activity [18]. IL-8 was shown to induce EMT in carcinomas through many signalling pathways, including the PI3K/Akt, NF-𝜅B, and Wnt signalling pathways [19]. Moreover, an inflammatory response activated by lipopolysaccharide (LPS) may also lead to EMT, as observed in a previous *in vivo* study [20].

### 2.2. Airway Inflammation and EMT-Induced Remodeling

Generally, most cases of asthma start in childhood and are associated with inhaled allergens such as house dust mites (HDMs), pollen, and animal dander [21]. Allergens are taken up and processed by dendritic cells (DCs), which present antigenic molecules to naive T helper cells. Thus, allergen-specific T cells are activated, and asthma develops. T helper type-2 (Th2) cells are stimulated by these allergens, which cause them to secrete cytokines such as IL-4, IL-5, and IL-13. Th17 cells, which produce IL-17A, IL-17F, and IL-22, are also known to modulate this disease.

Th2 cytokines, TGF-*β*, VEGF, and MMP9 are related to airway remodeling [20, 21]. These cytokines can interact with their respective receptors, activate Smad transcription factors, and cause changes in airway thickness [22]. It was also demonstrated that anti-IL-5 reduces the level of TGF-*β*1 expression in airway eosinophils and reduces the extent of airway remodeling, as assessed by bronchial biopsy [23]. The endogenous proinflammatory cytokines produced by Th2 lymphocytes associated with allergic diseases are mainly regulated by regulatory T lymphocytes [24].

Airway remodeling has been reported to be associated with airway inflammation in many studies. Th2 cytokines, TGF-*β*, vascular endothelial growth factor, and MMP9 are all related to airway remodeling [25, 26]. Patients with asthma exhibit thickened airway walls, increased mucous secretion, and inflammatory exudates [27]. It was reported that airway inflammation with eosinophil infiltration led to airway remodeling in mouse models of asthma [28]. During the airway remodeling process, eosinophils were shown to interact with mast cells and epithelial cells, as well as increase the release of TGF-*β*, cationic proteins, and cytokines.

Throughout the remodeling process, TGF-*β*/Smad signalling is considered to be the most important pathway. TGF-*β*1, which has a strong fibrogenic effect, is present in various tissues and cells as the most abundant transforming growth factor. This factor contributes to the development of EMT in alveolar epithelial cells and the induction of lung fibrosis [29]. This finding was confirmed in a previous study, and it was shown that anti-TGF-*β*1 treatment suppressed EMT in airway epithelial cells [30]. Furthermore, TGF-*β* was shown to upregulate the expression of *α*-smooth muscle actin and vimentin, suppress E-cadherin expression, and reduce the intercellular adhesion and polarity of epithelial cells. Notably, inflammation is known to modulate TGF-*β*1 expression in lung epithelial cells [31]. In addition, the function of TGF-*β* is enhanced by TNF-*α* [32].

The airway epithelium acts as a barrier against allergens from the environment due to the function of complete tight junctions. However, many allergens can disrupt tight epithelial junctions in patients with asthma, such as components of HDMs, cockroaches, and fungi. HDMs were shown to enhance the effect of TGF-*β*-induced EMT in bronchial epithelial cells and induce the expression of vimentin and fibronectin [33]. The defective epithelial cells induce danger signals that penetrate the epithelium to reach DCs [34].

Airway smooth muscle (ASM) cells contribute to airway inflammation and remodeling. ASM cells can be converted to proliferative and secretory cells by viruses and IgE. The proliferation of smooth muscle cells is also related to the production of matrix metalloproteinases [35].

Therefore, we believe that airway inflammation causes airway remodeling through the mesenchymal interactions between the epithelium and the lower layers and the dynamic interaction between cells and their released cytokines and mediators.

## 3. The Effect of Medicine on EMT in Airway Remodeling

According to the abovementioned reports, EMT is associated with epithelial cells, airway smooth muscle cells, eosinophils, mast cells, and the immune microenvironment, which together cause airway remodeling. Therefore, considerable progress must be made in research on treatments that target EMT to prevent airway remodeling.

### 3.1. The Effect of Herbal Medicines on EMT

Herbal medicines, which have been practised for thousands of years, are considered to be important for treating various diseases with few side effects [36]. Multicomponent and multitarget agents have attracted the interest of researchers [37]. In recent years, an increasing number of studies have shown that herbal formulas (Table 1) suppress airway remodeling by targeting EMT.

The most commonly used herb in Chinese medicine is the licorice (*Glycyrrhiza uralensis* Fisch (GanCao)). The modified KuShen–GanCao formula (mKG) was shown to suppress inflammation and pulmonary fibrosis and decreased the levels of IL-6, IL-17, and TGF-*β* in the lungs of mice with asthma [39, 40]. Huangqi–Fangfeng (Yupingfengsan) was reported to regulate the secretion of endothelial growth factor and TGF-*β*1, inhibit EMT changes, and ultimately prevent airway remodeling in mouse models with HDM-induced asthma [2]. Shenqi Cordyceps capsules have also been shown to reduce the inflammatory reaction and collagen deposition in the lung tissue of rats with pulmonary fibrosis induced by intratracheal instillation of bleomycin [38]. Yanghe Pingchuan granules were confirmed to attenuate asthma airway remodeling in a dose-dependent manner by reducing the expression of phosphoinositide 3-kinase (PI3K) and proliferating cell nuclear antigen (PCNA), blocking the PI3K/PKB signalling pathway, suppressing the abnormal proliferation of ASMCs, and alleviating the symptoms of kidney yang deficiency [41]. Soufeng Yuchuan decoction was reported to relieve airway remodeling and lung injury caused by mice with ovalbumin- (OVA-) induced asthma by downregulating the expression of TGF-*β*1 and VEGF [42]. The Pingchuan I formula was shown to treat the symptoms of allergic airway inflammation and airway remodeling in mice with asthma by decreasing the levels of PDGF-B and Erk1 [44]. Xiaoqinglong decoction was reported to have an inhibitory effect on airway remodeling in the lung tissues of mice with asthma by suppressing the expression of TGF-*β*1 and IL-13 [45]. Suhuang antitussive capsule (Suhuang) is a proprietary herbal medicine composed of nine herbs, including *Folium perillae*, *Herba ephedrae*, *Pheretima*, *Periostracum cicadae*, *Fructus arctii*, *Fructus schisandrae chinensis*, *Folium eriobotryae*, *Radix peucedani*, and *Fructus perillae*. Suhuang, which is widely used to treat asthma, suppresses inflammation and regulates immune function [50]. Lower doses of Suhuang can inhibit airway inflammation and remodeling in OVA-induced asthma by inhibiting IL-13 and TGF-*β*1 [43]. Huangqin (*Scutellaria baicalensis*) is frequently used to treat influenza, cancer, and chronic inflammatory diseases in the respiratory system [51]. Huangqin can attenuate airway remodeling by suppressing the levels of TGF-*β*1, MMP2, MMP9, and TIMP-1 in the PI3K/Akt/NF-𝜅B pathway [49].

In addition to herbal formulas, single herb formulations have also been shown to inhibit EMT in many studies. It was suggested that cultured Dong chong xia cao (*Cordyceps sinensis)* delayed fibrosis in airway epithelial cells by inhibiting the expression levels of phosphorylated (p)-Smad2, p-Smad3, and TGF-*β*1 and their receptors in the lungs of rats with COPD [46]. Fuling, Yiyiren, and Dongguazi have been shown to treat bleomycin-induced pulmonary fibrosis by reducing serum levels of TGF-*β*1 and TNF-*α* [47]. Danshen, an herb that promotes blood circulation and removes blood stasis, inhibits airway remodeling in rats with asthma by inhibiting the expression of TGF-*β*1 and MMP9 [48].

### 3.2. The Effect of Specific Herbal Compounds on EMT

In recent years, an increasing number of studies have shown that various specific compounds found in herbs (Table 2) suppress airway remodeling by targeting EMT. Glycyrrhizin, an active constituent of licorice, was shown to reduce the thickness of the airway epithelium, basement membrane, and subepithelial smooth muscle layers [67]. It has been shown to ameliorate airway remodeling via the TGF-*β*1/Smad signalling pathway in mice [52].

Diosmetin (3,5,7-trihydroxy-4-methoxyflavone), a natural flavonol-type flavonoid found in citrus fruits, olive leaves, and other plants, has multiple biological activities, such as the regulation of body inflammation, antioxidant effects, and antitumour effects [68]. In a study on HBE cells, diosmetin inhibited the development of EMT and the production of intracellular reactive oxygen species (ROS) induced by TGF-*β*1, altered cell migration, and reversed the expression of N-cadherin, E-cadherin, and *α*-smooth muscle actin. It also suppressed TGF-*β*1-induced phosphorylation of the PI3K/Akt and MAPK pathways in HBE cells [69]. Diosmetin was also reported to prevent the production of intracellular ROS and suppress the expression of *α*-SMA, MMP9, and TGF-*β*1 in mouse models of chronic asthma [53, 54].

Sinomenine is a natural alkaloid derived from the roots and stems of the climbing plant *Sinomenium acutum*. It was demonstrated that sinomenine suppresses the expression of inflammatory mediators in rats [70]. The inhibitory effect of sinomenine on airway remodeling was also demonstrated in an animal model of asthma, and the levels of TGF-*β*1 were reduced [55].

Emodin is a widely used compound isolated from the rhizomes of *Rheum palmatum* and has antitumour and anti-inflammatory activities [71]. It was found that emodin inhibited TGF-*β*1-induced EMT by regulating the notch signalling pathway, reducing notch-1 nuclear translocation, and inhibiting the proliferation of rat type-II alveolar epithelial cells (RLE-6TN) in a concentration-dependent manner [56]. Emodin was also shown to reduce collagen I, *α*-SMA, and vimentin, as well as promote the expression of E-cadherin.

Amygdalin is an active component of the bitter almond that has a wide range of pharmacological effects such as the inhibition of tissue fibrosis. It was found that amygdalin attenuated EMT in both mice with COPD and BEAS‐2B cells. Amygdalin may also suppress TGF‐*β*1 expression and the phosphorylation of Smad2/3 in the TGF‐*β*/Smad pathway [57].

Hexamethoxy flavanone-o-[rhamnopyranosyl-(1 ⟶ 4)-rhamnopyranoside] (HMFRR) is a flavonoid glycoside isolated from *Murraya paniculata* (L.). In A549 cells, HMFRR effectively suppressed the levels of integrin *β*1, EGFR, COX-2, MMP2, MMP9, and EMT markers, and it upregulated E-cadherin by interrupting the STAT3/NF-*κ*B/COX-2 and EGFR/PI3K/Akt signalling pathways [58].

Tetrandrine is a common bisbenzylisoquinoline alkaloid extracted from the root of *Stephania tetrandra* S. Moore [72]. In respiratory diseases, it was found that tetrandrine alleviated the inflammatory response by reducing the secretion of IL-2, IL-4, and IFN-c in patients with asthma [73]. It was also reported that tetrandrine attenuated OVA-induced airway remodeling in rats by inhibiting the expression of MMP9 and TGF-*β*1 [59]. Tetrandrine also promoted Nrf-2 nuclear transcription and suppressed TGF-*β*1-induced proliferation in ASM cells by relieving oxidative stress.

Naringenin, which is found in grapefruits and tomatoes, is an important component of the Qingfei Tongluo formula that exhibits a wide range of pharmacological properties. There is evidence that naringenin inhibits liver fibrosis by regulating TGF-*β* signalling [74]. Naringenin also protects against stress-induced autophagy and inhibits lung damage caused by oxidative stress [75, 76]. Moreover, naringenin was shown to have a significant therapeutic effect on *Mycoplasma pneumoniae*-induced lung injury in mice by inhibiting pulmonary fibrosis and inhibiting the secretion of inflammatory cytokines such as IL-6, IL-1*β*, TNF-*α*, and TGF-*β* [60].

Icariin, a major active component of Epimedium, has been reported to improve cardiovascular function and induce tumour cell differentiation. It also plays a therapeutic role in the management of airway remodeling and was shown to inhibit the proliferation of ASM cells via the MAPK/Erk pathway in an OVA-induced asthma model [61].

Nodakenin is a furocoumarin glucoside found in the roots of *P. decursivum* Maxim. It has been commonly used to treat patients with asthma and chronic bronchitis for thousands of years without any side effects. It was reported that nodakenin markedly inhibited airway inflammation, airway remodeling, and smooth muscle hypertrophy by decreasing the levels of IL-4, IL-5, IL-13, and MMP2/9, as well as reducing NF-𝜅B DNA-binding activity in lung tissue [62].

Dioscin, a kind of steroidal saponins extracted from some medicinal plants, has multiple medicinal effects including anti-inflammatory and anticancer effects. It was reported that dioscin restrained chronic asthmatic mice by altering TGF-*β*1/Smad2/3 and Akt signalling pathways and reversed TGF-*β*1-induced EMT in 16HBE cells [63].

Galangin is a natural flavonol with potential anti-inflammatory properties that may attenuate airway remodeling in ova-induced mice by inhibiting TGF-*β*1, MMP9, and VEGF [64].

Artesunate is a semisynthetic single compound isolated from the plant *Artemisia annua*. Artesunate may decrease inflammation and attenuate airway remodeling in asthmatic mice via the MAPK signalling pathway [65].

### 3.3. The Effect of Specific Cytokines or Amino Acids on EMT

IL-24 was upregulated in the nasal secretions and sputum of asthma patients and could induce EMT during airway remodeling (Table 3). However, IL-37 was proven to alleviate IL-24-induced EMT in asthmatic airway remodeling via the Erk1/2 and STAT3 pathways [77]. Carbocisteine (S-carboxymethylcysteine, SCMC) was reported to inhibit chronic obstructive pulmonary disease. Its alleviating effects have also been implicated in mice with asthma, and it can inhibit TGF-*β*1 expression and collagen fibre deposition in airway tissues [78]. The recombinant pyrin domain protein was demonstrated to attenuate airway remodeling in asthmatic mice through the TGF-*β*1/Smad and Jagged1/Notch1 signalling pathways [79]. Empagliflozin is a selective inhibitor of Na+ -glucose cotransporter-2 with anti-inflammatory and antifibrotic effects. It has been demonstrated that empagliflozin inhibits autophagy and has antiasthmatic effects and antiremodelling properties in mice with allergic asthma [80].

### 3.4. The Effect of Specific MicroRNAs or Vitamins on EMT

MicroRNAs, which are small noncoding RNAs, are involved in a variety of cellular processes that regulate gene expression. In recent years, it has been proven that microRNAs may suppress EMT in the airway in asthmatic mice through a target gene (Table 4). miR-124-3p attenuates inflammation and EMT in asthma mouse models by targeting S100A4 and suppressing the TGF-*β*/Smad2 signalling pathway [81]. miR-506 inhibits the airway inflammatory response and remodeling by mediating Wnt/*β*-catenin signalling and targeting polypyrimidine tract-binding protein 1 [82]. Vitamin D is commonly known for its ability to inhibit airway remodeling, and it was reported to inhibit vimentin and TGF expression [83].

## 4. Discussion

Airway inflammation and airway remodeling are the main features of various lung diseases. Many factors in epithelial cells have been identified as novel markers of the dysregulation of epithelial-mesenchymal signalling, which is present in all cases of asthma, including severe cases [84, 85]. Epithelial barrier dysfunction, which is induced by injury and repair in chronic inflammation, is associated with a persistent dedifferentiation program of complex aetiology.

After recurrent asthma attacks, airway remodeling occurs and lung function is damaged. Airway fibrosis is the pathological characteristic of severe asthma [86]. There is currently no particularly effective treatment for pulmonary fibrosis. Corticosteroids are not recommended as drugs to treat pulmonary fibrosis due to their cytotoxicity, although they have anti-inflammatory effects [87]. Other drugs, including bosentan, a dual endothelin receptor antagonist, have been proven to have poor therapeutic effects on pulmonary fibrosis in blinded randomized trials. Some metabolic pathway inhibitors, including AM966, fasudil, and simtuzumab, may have effects on pulmonary fibrosis, but they still need further validation [66, 88–91]. There are 2 approved antifibrotic drugs, nintedanib and pirfenidone, for IPF and pulmonary fibrosis of secondary origin, which can slow but not halt disease progression [91]. These therapies do not inhibit airway remodeling by inhibiting EMT.

This review focused on the treatment of airway remodeling from the perspective of EMT. EMT is an important mechanism of airway remodeling and is related to many signalling pathways and molecules, such as TGF-*β*/Smad, NF-*κ*B, PI3K/Akt, EGFR, and MMP2. These molecules have emerged as the main targets for the treatment of airway remodeling. The TGF-*β*/Smad signalling pathway is the most important pathway involved in EMT progression and has been studied as a target for the prevention of EMT in airway epithelial cells. This study reviewed the current research on airway remodeling drugs and their effects on the EMT pathway. Various drugs, including herbal formulations, specific herbal compounds, cytokines, amino acid or protein inhibitors, microRNAs, and vitamins, may suppress airway remodeling by inhibiting EMT-related pathways. Research on herbs and their compounds accounts for a significant portion of research in this field. Many herbs have shown clinical effects related not only to anti-inflammatory effects but also to airway remodeling that targets EMT. However, their main components and mechanisms are unclear. Further research is needed to determine the exact molecular mechanisms by which herbal compounds affect EMT. The target receptors of the active compounds of herbs must also be identified. In addition, although targets for some compounds have been identified, the relationship between the proteins and the compounds requires further experiments *in vitro* and *in vivo,* and there must be clinical trials of already licenced drugs.

## 5. Conclusion

The dysregulation of EMT in airway cells is associated with airway remodeling and pulmonary fibrosis progression. T helper cells are stimulated by allergens, which leads to the release of cytokines such as IL-4, IL-5, and IL-13. Cytokines act as mediators, interact with their receptors, and activate transcription factors to induce EMT. Various herbal compounds, specific cytokines or amino acids, some microRNAs, and vitamins can suppress airway remodeling by EMT through the suppression of pathways involving TGF-*β*1 or other cytokine-related pathways.

## Figures and Tables

**Table 1:** Herbal medicine that acts on EMT in different respiratory symptoms.

Herbs	Formal name	English name	Respiratory symptoms	EMT-related target
Huangqi–Fangfeng [2]	Huang qi (*Astragalus membranaceus* (*Fisch.*) *Bge. var. mongholicus* (*Bge*.) *Hsiao*)	Astragali radix	HDM-induced asthma	TGF-*β*1
	Fang feng (*Saposhnikovia divaricata* (*Turcz.*) *Schischk*)	Saposhnikoviae radix		

Shenqi Cordyceps capsules [38]	Xi yang shen (*Panax quinquefolium* L)	Panacis quinquefolii radix	Pulmonary fibrosis in rats induced by intratracheal instillation of bleomycin	*α*-SMA
	Dong chong xia cao (*Cordyceps sinensis* (BerK.) Sacc)	Cordyceps		
	*San qi* (*Panax notoginseng* (Burk.) F. H. Chen)	Notoginseng radix et rhizoma		

KuShen–GanCao formula [39, 40]	Kushen (*Sophora flavescens* Ait)	Sophorae flavescentis radix	Pulmonary fibrosis in rats	IL-6, IL-17, TGF-*β*1
	Gancao (*Glycyrrhiza uralensis* Fisch.)	Glycyrrhizae Radix et rhizoma		

Yanghe Pingchuan [41]	Shu di (*Rehmannia glutinosa* Libosch.)	Rehmanniae radix praeparata	Egg albumin-induced asthmatic rat	PI3K, PCNA
	Yin yanghuo (*Epimedium brevicomu* Maxim.)	Epimedii folium		
	Danggui (*Angelica sinensis* (oliv.) Diels)	Angelicae sinensis radix		
	Mahuang (*Ephedra sinica* Stapf)	Ephedrae herba		
	Zi shi ying (*Fluorite*)	Fluoritum		
	Rougui (*Cinnamomum cassia* Presl)	Cinnamomi cortex		
	Baijiezi (*Sinapis alba* L)	Sinapis semen		
	Lujiaopian (*Cervus elaphus* Linnaeus)	Cervi cornu		
	Wuweizi (*Schisandra chinensis* (*Turcz.*) Baill)	Schisandrae chinensis fructus		
	Taoren (*Prunus persica* (L.) Batsch)	Persicae semen		
	Zaojiao (Gleditsia sinensis)	Gleditsiae sinensis fructus		

Soufeng Yuchuan decoction [42]	Dangshen (*Codonopsis pilosula* (*Franch.*))	Codonopsis radix	OVA-induced asthma in mice	TGF-*β*1, VEGF
	Honghua (*Carthamus tinctorius* L)	Carthami flos		
	Shanzha (*Crataegus pinnatifida Bge.*)	Crataegi fructus		
	Mahuang (*Ephedra sinica Stapf*)	Ephedrae herba armeniacae semen		
	Xingren (*Prunus armeniaca* L. *var. ansu Maxim*)	Amarum		
	Juluo (*Citrus tangerina Hort.et Tanaka C.erythrosa Tanaka*)	Citrus reticulata Blanco		
	Laifuzi (*Semen Raphani*)	Radish Seed		
	Pipaye (*Eriobotrya japonica* (*Thunb.*) *Lindl.*)	Folium eriobotryae		
	Chantui (*Cryptotympana pustulata Fabricius*)	Cicadae Periostracum		
	Dilong (*Pheretima aspergillum*)	Pheretima		
	Bai jiang can (*Bombyx mori Linnaeus*)	Batryticatus		
	Meng shi	Lapis chloriti		
	Gan cao (*Glycyrrhiza uralensis Fisch.*)	Glycyrrhizae Radix et rhizoma		

Suhuang antitussive capsule [43]	Mahuang (*Ephedra sinica* Stapf)	Ephedrae herba	OVA-induced asthma	IL-13, TGF-*β*1
	Zisuye (*Perilla frutescens* (L.) Britt)	Perillae Folium		
	Zi su zi (*Perilla frutescens* (L.) Britt)	Perillae fructus		
	Dilong (*Pheretima aspergillu*)	Pheretima		
	Pipaye (*Eriobotrya japonica* (Thunb.) Lindl)	Eriobotryae folium		
	Chantui (*Cryptotympana pustulata* Fabriciu)	Cicadae Periostracum		
	Qianhu (*Peucedanum praeruptorum* Dunn)	Peucedani Radix		
	Niubangzi (*Arctium lappa* L)	Arctii Fructus		
	Wuweizi (*Schisandra chinensis* (*Turcz.*) Baill)	Schisandrae chinensis fructus		

Pingchuan I formula [44]	Xuan fu hua (*Inula japonica Thunb*)	Inulae flos	Asthma in mice	PDGF-B and Erk1
	Dai zhu shi (*Haematite*)	Haematite		
	Bai jie zi (*Sinapis alba* L.)	Sinapis semen		
	Suzi (*Perilla frutescens* (*L.*)*Britt.*)	Perillae fructus		
	Tinglizi (*Descurainia sophia* (*L.*) *Webb*, *ex Prantl*)	Descurainiae semen lepidii semen		
	Banxia (*Pineilia ternata* (*Thunb.*) *Breit*)	Pinelliae rhizoma		
	Huangqin (*Scutellaria baicalensis Georgi*)	Scutellariae radix		
	Dilong (*Pheretima aspergillum^. Perrier*)	Pheretima		
	Gangban (*Polygonum perfoliatum* L.)	Polygoni perfoliati herba		

Xiaoqinglong decoction [45]	Ma huang (*Ephedra sinica* Stapf)	Ephedrae herba	OVA-induced asthma in mice	TGF-*β*1, IL-13
	Bai Shao (*Paeonia lactiflora* Pall)	Paeoniae radix alba		
	Xi xin (*Asarum heterotropoides fr. Schmidt var. mandshuricum* (Maxim)・Kitag)	Asari radix et rhizoma		
	Gan jiang (*Zingiber officinale* Rose)	Zingiberis rhizoma		
	Zhigancao (*Glycyrrhiza uralensis* Fisch)	Glycyrrhizae radix et rhizome		
	Guizhi (*Cinnamomum cassia* Presl)	Cinnamomi ramulus		
	Wuweizi (*Schisandra chinensis* (Turcz.) Baill)	Schisandrae chinensis fructus		
	Ban xia (Pinellia ternata (Thunb.) Breit)	Pinelliae rhizoma		

Dong chong xia cao [46]	Dong chong xia cao (*Cordyceps sinensis*)	Cordyceps	Rats with COPD	TGF-*β*1/Smad2, Smad3
Fuling [47]	Fuling (*Poria cocos* (Schw)Wolf)	Poria	Bleomycin-induced asthmatic rat	TGF-*β*1, TNF-*α*
Yiyiren [47]	Yiyiren (*Coix lacryma-jobi* L. var. mayuen (Roman.)Stap)	Coicis semen	Bleomycin-induced asthmatic rat	TGF-*β*1, TNF-*α*
Dongguazi [47]	Dongguazi (*Benincasa hispida* (Thunb.))	Winter melon seeds	Bleomycin-induced asthmatic rat	TGF-*β*1, TNF-*α*
Danshen [48]	Danshen (*Salvia miltiorrhiza* Bge)	Radix salvia miltiorrhizae	Bleomycin-induced asthmatic rat	TGF-*β*1, TNF-*α*
Huangqin [49]	Huangqin (*Scutellaria baicalensis* Georgi)	Scutellariae radix	Rats with cigarette smoke-induced COPD	PI3K/Akt/NF-*κ*B

**Table 2:** Herbal compounds that act on EMT in different respiratory symptoms.

Compounds	Model	EMT-related target
Glycyrrhizin [52]	OVA-induced asthma in mice	TGF-*β*1/Smad
Diosmetin [53, 54]	TGF-induced HBE16 cells	PI3K/Akt, MAPK
Sinomenine [55]	OVA-induced asthma in mice	TGF-*β*1
Emodin [56]	Rat type-II alveolar epithelial cells (RLE-6TN)	Notch-1, collagen I, *α*-SMA
Amygdalin [57]	COPD mice and BEAS-2B cells exposed to smoke	TGF-*β*/Smad3,
Hexamethoxy flavanone-o-[rhamnopyranosyl-(1,4)-rhamnopyranoside (HMFRR) [58]	A549	EGFR, COX-2, MMP-2, MMP-9, STAT3/NF-*κ*B/COX-2, EGFR/PI3K/Akt
Tetrandrine [59]	OVA-induced asthma in male Wistar rats	TGF-*β*1/Nrf-2/HO-1
Naringenin [60]	Mycoplasma pneumoniae (MP)-induced lung injury	IL-6, IL-1*β*, TNF-*α*, and TGF-*β*
Icariin [61]	OVA-induced asthma in mice	MAPK/Erk
Nodakenin [62]	OVA-induced asthma in mice	IL-4, IL-5, IL-13, MMP2/9, and NF-*κ*B
Dioscin [63]	OVA-induced asthma in mice TGF-induced HBE16	TGF-*β*/Smad
Galangin [64]	OVA-induced asthma in mice	TGF-*β*1, MMP9, VEGF
Artesunate [65]	OVA-induced asthma in mice	MAPK
Ginkgolic acid [66]	Pulmonary fibrosis in mice	Smad4

**Table 3:** Specific cytokines or amino acids involved in EMT in different respiratory symptoms.

Compounds	Model	EMT-related target
IL-37 [77]	House dust mite-induced asthmatic mice	Erk1/2 and STAT3
Carbocisteine [78]	OVA-induced asthmatic mice	TGF-*β*1
The recombinant pyrin domain protein [79]	Asthmatic mice	TGF-*β*1/Smad and Jagged1/Notch1
Empagliflozin [80]	Asthmatic mice	Autophagy

**Table 4:** Specific microRNAs or vitamins involved in EMT in different respiratory symptoms.

Compounds	Model	EMT-related target
miR-124-3p [81]	Asthmatic mouse models	TGF-*β*/Smad2
miR-506 [82]	TGF-*β*1-induced ASMCs cells	Wnt/*β*-catenin
Vitamin D [83]	Calcitriol, beclomethasone 17-propionate, montelukast sodium, LTD4 and TGF-*β*-induced HFL1 cells	Vimentin, TGF

## Abbreviations

EMT: Epithelial-mesenchymal transition
COPD: Chronic obstructive pulmonary disease
DCs: Dendritic cells
Th2: T helper type-2
PI3K: Phosphoinositide 3 kinase
TGF-*β*1: Transforming growth factor-beta 1
PDGF: Platelet-derived growth factor
CTGF: Connective tissue growth factor
FGF: Fibroblast growth factor
VEGF: Vascular endothelial growth factor
OVA: Ovalbumin
HDM: House dust mite.
